# The Hepatitis E Virus Open Reading Frame 2 Protein: Beyond Viral Capsid

**DOI:** 10.3389/fmicb.2021.739124

**Published:** 2021-10-07

**Authors:** Zhaobin Zhou, Yinqian Xie, Chunyan Wu, Yuchen Nan

**Affiliations:** ^1^Department of Preventive Veterinary Medicine, College of Veterinary Medicine, Northwest A&F University, Yangling, China; ^2^Scientific Observing and Experimental Station of Veterinary Pharmacology and Diagnostic Technology, Ministry of Agriculture, Yangling, China; ^3^Shaanxi Animal Disease Prevention and Control Center, Xi’an, China

**Keywords:** hepatitis E virus, HEV-ORF2 protein, capsids, innate immunity, subunit vaccine, quasi-enveloped virion

## Abstract

Hepatitis E virus (HEV) is a zoonotic pathogen causing hepatitis in both human and animal hosts, which is responsible for acute hepatitis E outbreaks worldwide. The 7.2 kb genome of the HEV encodes three well-defined open reading frames (ORFs), where the ORF2 translation product acts as the major virion component to form the viral capsid. In recent years, besides forming the capsid, more functions have been revealed for the HEV-ORF2 protein, and it appears that HEV-ORF2 plays multiple functions in both viral replication and pathogenesis. In this review, we systematically summarize the recent research advances regarding the function of the HEV-ORF2 protein such as application in the development of a vaccine, regulation of the innate immune response and cellular signaling, involvement in host tropism and participation in HEV pathogenesis as a novel secretory factor. Progress in understanding more of the function of HEV-ORF2 protein beyond the capsid protein would contribute to improved control and treatment of HEV infection.

## Introduction

Hepatitis E virus (HEV), a positive-sense and single-stranded RNA virus, is now classified as the only member of the family *Hepeviridae* (Smith et al., 2014). HEV viral particles can be either enveloped or non-enveloped, which has been recently defined as the quasi-enveloped viral form (Nan et al., 2017b). Generally, HEV virions obtained from fecal samples of HEV patients are non-enveloped form as spherical particles with a diameter of 27–34 nm (Meng, 2016). Contrastingly, HEV virions collected from the serum of HEV patients contain a lipid envelope similar to classic enveloped virus. HEV infection in the general population generally leads to self-limiting hepatitis with a mortality rate about 0.5–3%. However, in pregnant women, HEV infection has an extremely high mortality rate, which could be potentially up to 30% if the infection occurs in the third trimester of gestation (Jameel, 1999).

Ever since the fecal-oral route for HEV transmission has been documented, HEV has been proposed as a public health concern only in developing countries until the emergence of zoonotic HEV infections in industrialized countries (Christensen et al., 2008; Dalton et al., 2008; Meng, 2013; Pavio et al., 2015). Currently, Hepatitis E cases have been frequently reported in industrialized countries, and inter-species transmission of HEV from zoonotic reservoirs to humans has been proposed as the major factor for sporadic HEV cases in corresponding countries (Pavio et al., 2015; Hazards et al., 2017). Besides zoonosis, another unique feature of HEV is its variable pathogenesis and disease forms of clinical symptoms, such as chronic infection in immunocompromised individuals and extrahepatic invasion during chronic HEV infection (Kamar et al., 2011, 2012; Hoofnagle et al., 2012; Grewal et al., 2014; van Eijk et al., 2014; Dalton et al., 2016; Geng et al., 2016). Nevertheless, our understanding of HEV remains far from enough.

The HEV-ORF2 protein, originally identified as the major component for HEV capsid, was presumably considered to bind with cellular receptors to mediate HEV infection of susceptible cells both *in vivo* and *in vitro* (Nan et al., 2017b). Therefore, this function makes HEV-ORF2 a perfect target for subunit vaccine development. However, in recent years, novel roles of the HEV-ORF2 protein played during viral replication have been identified and HEV-ORF2 exists as different forms other than as capsids in infected cell, such as the secreted form of the full-length ORF2 and cleaved forms of ORF2 proteins. These observations further indicate that the HEV-ORF2 protein performs multiple roles besides solely acting as the structure component to build virion. In this review, we systematically summarize the current literature regarding HEV-ORF2 protein function and discuss in detail to provide new insights into host tropism by HEV-ORF2 and the novel functions of the secreted form of HEV-ORF2.

## Molecular Biology and Zoonosis of Hepatitis E Virus

**FIGURE 1:**
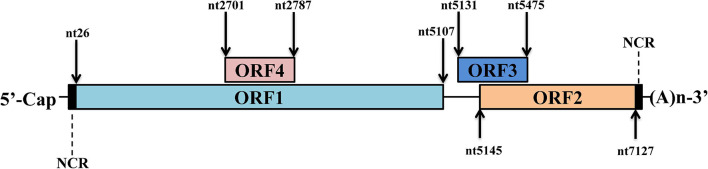
Schematic illustration of HEV genome organization. Schematic illustration of HEV genome and location of ORFs. The ORF1 (nt 26–5107) is labeled above the genomic RNA box. ORF2 (nt 5145–7127) and ORF3 (nt 5131–5475) are encoded by the same subgenomic RNA. The numbers above or below the RNA boxes indicate nucleotide numbers of the cDNA of HEV-1 Sar55 strain (GenBank accession number: AF444002).

The full genome of mammalian HEV is an mRNA-like structure and near 7.2 kb in length. As a typical positive-stranded RNA virus, the HEV genome is capped and poly-adenylated at its 5′ end and 3′ end, respectively (Ahmad et al., 2011). Up to date, three well-characterized open reading frames (ORFs) have been identified from its genome (Figure 1). the HEV-ORF1 protein is translated from the mRNA-like genome and encodes all viral replicases required for viral RNA replication (Tam et al., 1991; Tsarev et al., 1992). HEV-ORF2 and ORF3 are overlapped with each other and translated from viral sub-genome mRNA generated during virus replication (Graff et al., 2006). The protease-cleaved shorter form of the HEV-ORF2 protein forms the viral capsid and is the major component of building the HEV virion, whereas the HEV-ORF3 protein appears to be a class I viroporin essential for the release of HEV virion particles and the biogenesis of the quasi-enveloped virion (Mori and Matsuura, 2011). In addition to these ORFs, an additional ORF4 (embedded within ORF1) has been identified recently, and HEV-ORF4 protein expression is driven by an upstream internal ribosome entry site (IRES)-like sequence (Nair et al., 2016). However, it appears that ORF4 is not conserved among HEV genotypes and is only present in HEV-1 (Nair et al., 2016).

Currently, available HEV isolates are highly diverse, and the latest categorization of HEVs divides all HEVs and HEV-like virus isolates into two genera under the family name of *Hepeviridae* as either *Orthohepevirus* (covering all virus isolates with origins from mammalian and avian species) or *Piscihepevirus* (covering only viral isolates with origins from cutthroat trout). Under the *Orthohepevirus* genus, different virus isolates are further categorized into species according to their hosts and sequences (Smith et al., 2014). HEV genotypes infecting humans include two anthropotropic genotypes (HEV-1 and HEV-2) and two zoonotic genotypes (HEV-3 and HEV-4) and are classified under the species *Orthohepevirus A* (Nan et al., 2017b), while species B, C, and D of the *Orthohepevirus* genus include virus isolates originating from other non-human mammalian hosts (Smith et al., 2014; Nan et al., 2017b). However, it is notable that HEV-7 (camel origin) could be a potential zoonotic HEV genotype similar to HEV-3 and 4, as an HEV strain isolated from an organ transplant recipient was closely related to HEV-7 (Smith et al., 2014; Lee et al., 2016; Woo et al., 2016), suggesting there might be more zoonotic genotypes of HEV than previously characterized.

## Basic Functions of Proteins Encoded by Hepatitis E Virus

### Open Reading Frame 1 Protein

The ORF1 protein as HEV replicase, is the largest HEV protein and can be directly translated from HEV genome (Tsarev et al., 1992). It remains inconclusive whether the HEV-ORF1 protein acts as a single protein to carry out all putative functions as viral replicases or if it is a polyprotein precursor requiring further cleavage by host or viral proteases to generate independent units (Parvez, 2013, 2017; Paliwal et al., 2014; Nan and Zhang, 2016). Based on a protein homology comparison, at least five enzymatic domains are presented in the HEV-ORF1 protein, including methyltransferase, papain-like cysteine protease, RNA helicase, and RNA-dependent RNA polymerase domains as well as a macro domain (Ahola and Karlin, 2015; Nan and Zhang, 2016). Besides these enzymatic domains, there are Y domains (based on similarity to the Y domain of the rubella virus) presenting downstream of the methyltransferase domain and a hypervariable region linking the papain-like cysteine protease and macro domains (Koonin et al., 1992). The methyltransferase domain and Y domain are thought to assemble together as viral RNA capping enzyme (Ahola and Karlin, 2015; Nan and Zhang, 2016). Conversely, the macro domain (homolog with the non-histone region of the macroH2A histone) is generally identified as the flanking region along with the papain-like cysteine protease domain in many RNA viruses such as coronavirus (Gorbalenya et al., 1991; Koonin et al., 1992). The macro domain of HEV appears to be a catalytic homolog to the ADP-ribose-1′-monophosphatase of coronaviruses, which is essential for viral replication (Parvez, 2015). The hypervariable region is composed of two parts: the proline-rich domain and the hypervariable domain. The proline-rich domain was proposed to act as a hinge that forms an unstable tertiary structure (Koonin et al., 1992; Tsai et al., 2001; Dosztanyi et al., 2006; Dunker et al., 2008), whereas the hypervariable domain is considered to be an intrinsically disordered region (IDR), which is highly susceptible to extensive gene segment insertions or deletions (Pudupakam et al., 2009, 2011; Purdy, 2012; Purdy et al., 2012), even gene segment originating from hosts. An example of this is the HEV-3 Kernow-C1 p6 strain which contains a 174-nt insertion of the human ribosomal protein S17 (Shukla et al., 2011).

### Open Reading Frame 2 Protein

**FIGURE 2:**
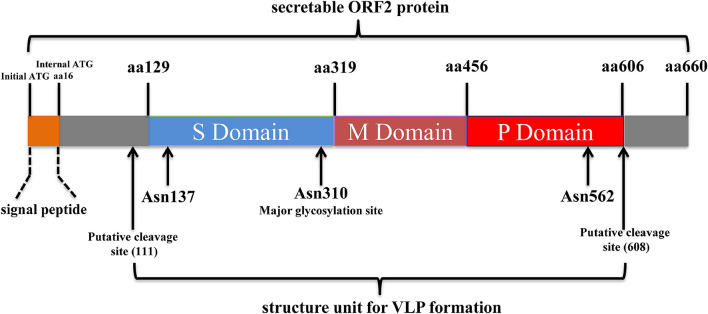
Schematic illustration of structure and functional domains of HEV-ORF2 protein. The capsid protein has three domains designated as Shell (S), Middle (M), and Protruding (P) domain. The S domain is responsible for forming the *T* = 1 icosahedral shell of virion which contains 60 copies of capsid protein. The M domain binds tightly to the S domain and interacts with two 3-fold-related M domains to form a surface plateau. The P domains are tightly associated as a dimeric spike which is responsible for binding with susceptible cells.

As the major virion component, HEV-ORF2 was initially considered to encode the capsid protein (Robinson et al., 1998). However, in recent years it was demonstrated that HEV-ORF2 proteins present as different forms with multiple functions rather than solely acting as the viral capsid (Montpellier et al., 2018; Ankavay et al., 2019). The full-length ORF2 protein carries N-terminally linked glycans at three different Asn sites (aa137, 310, and 562) as well as an endoplasmic reticulum (ER)-directing signal peptide in its N-terminus (Jameel et al., 1996). The signal peptide of the full-length ORF2 earmarked ORF2 for subsequential glycosylation and secretion to extracellular space (Yin et al., 2018). The glycosylated full-length ORF2 protein exists as a dimer and demonstrates a difference in antigenicity with the HEV capsid, as epitopes predicted to bind to the cell receptor are lost. Therefore, the secreted full-length ORF2 protein does not block HEV cell entry but inhibits antibody-mediated neutralization to HEV (Yin et al., 2018). Besides the full-length HEV-ORF2 protein, an alternative translation of the ORF2 protein can be initiated at an internal start codon (aa16 of the full-length ORF2) downstream of the ER-directing signal peptide to generate the HEV capsid protein (Yin et al., 2018). This internal translated ORF2 protein undergoes proteolytic processing to remove part of its N-terminal and C-terminal aa residues in order to generate the structure unite for mature capsid. Compared to the full-length ORF2 protein, the HEV capsid protein lacks 111 aa and 52 aa residues at its N-terminal and C-terminal, respectively. The recombinant HEV capsid can form virus-like particles (VLPs) when expressed in insect cells (Li et al., 1997, 2005c). Both conformational and linear neutralizing epitopes were identified from the HEV capsid (Gu et al., 2015; Tang et al., 2015), and a recombinant subunit vaccine based on a truncated HEV capsid has been licensed in China (Nan and Zhang, 2016). In addition to the two above ubiquitous forms of HEV-ORF2 proteins, a recent study demonstrated that a truncated form of ORF2 (potentially cleaved by unknown protease) could be detected in HEV-infected cells using mass spectrometry method and may be secreted into the extracellular environment similar to that of full length ORF2 protein (Montpellier et al., 2018). However, the exact initial aa site at the N-terminal of this truncated form of ORF2 remains unknown since mass spectrometry analysis only detected the serine residue in 102aa of truncated form of ORF2 (Ankavay et al., 2019). Meanwhile, it is also unclear how this type of ORF2 protein could be secreted extracellularly since the signal peptide was cleaved. The function of this cleaved ORF2 protein and its biological significance in the HEV lifecycle requires further investigation. A schematic illustration of structure and functional domain of HEV-ORF2 based on the sequence of prototype strain HEV-1 Sar55 was listed as Figure 2.

As viral capsid protein, the major function of HEV-ORF2 is to mediate the attachment and infection of susceptible cells by HEV. For the naked HEV particles, the capsid protein is believed to bind to a cellular receptor to initiate viral entry. Structure and sequence analyses suggest that the putative binding motif of the capsid protein is conserved among all four major human HEV genotypes (Guu et al., 2009), whereas HEV capsids contain β-barrel fold structures which are predicted to have polysaccharide-binding sites and appear to be involved in both cell-receptor binding and capsid disassembly (He et al., 2008; Zhao et al., 2015). Early report employing HEV-VLPs suggested that attachment of HEV-VLP to cells surface depends on heparin sulfate proteoglycans (HSPGs) and pre-incubation of HEV with heparan sulfate blocks HEV infectivity (Kalia et al., 2009; Yin et al., 2016), whereas sequential research identified asialo-glycoprotein receptor (ASGPR) and retinol-binding protein 4 (RBP4) as putative HEV capsids interacting receptors (Shen et al., 2011; Zhang et al., 2016). Meanwhile, a recent study based on avian HEV suggested that organic anion-transporting polypeptide 1A2 (OATP1A2) may be an avian HEV-specific receptor (Li et al., 2019), but whether mammalian counterpart of OATP1A2 acts as potential receptor for mammalian HEV requires further investigation.

After binding with its receptor, naked HEV particles are internalized *via* a dynamin-2, clathrin, and membrane cholesterol-dependent pathway (Kapur et al., 2012; Holla et al., 2015). Meanwhile, Heat-shock protein 90 (HSP90), Heat shock cognate protein 70 (HSC70) and Glucose-regulated protein 78 (Grp78) are found to be involved in intra-cellular transport for HEV (Kalia et al., 2009; Zheng et al., 2010; Yu et al., 2011; Cao and Meng, 2012), whereas HSP90-specific inhibitor (geldanamycin) blocks the intracellular transport of the HEV-VLP without affecting its entry (Zheng et al., 2010). However, quasi-enveloped HEV particles enter cells *via* a distinct pathway that involves in degradation of the lipid membrane in the lysosome which involves small GTPases Rab5 and Rab7 (Yin et al., 2016). After internalization into permissive cells, the HEV capsid is uncoated by unknown mechanism, followed by HEV-ORF1 translation to start the HEV-RNA replication cycle.

### Open Reading Frame 3 Protein

The HEV-ORF3 protein is translated from the same sub-genomic mRNA as that of HEV-ORF2 and partially or completely overlaps with ORF2 based on HEV species. The HEV-ORF3 protein is 113 aa residues in length. Currently, available reports favor the concept that the ORF3 protein plays indispensable roles in the release of HEV virions from infected cells and involves in the biogenesis of so called “quasi-enveloped” HEV virions (Hurley, 2010; Nagashima et al., 2011a, b, 2014; Feng et al., 2014; Yin et al., 2016). Two functions have been proposed for HEV-ORF3 protein. On the one hand, HEV-ORF3 acts as an ion channel and shares key structural features with class I viroporin. Viroporin is generally required for virion release from infected cells for many viruses. The viroporin function of HEV-ORF3 could be replaced by the influenza virus M2 protein, a well-defined viroporin (Ding et al., 2017). On the other hand, the PSAP motif located at aa 95–98 of the HEV-ORF3 protein is indispensable for the formation of membrane-associated HEV particles (the quasi-enveloped virions), a process requiring the association of ORF3 with lipids, thus potentially contributes to budding of quasi-enveloped HEV (Nagashima et al., 2010, 2011a). This observation is further evidenced by interaction between tumor susceptibility gene 101 (Tsg101) and HEV-ORF3 protein (Nagashima et al., 2011a, 2014). The Tsg101 is an key component of endosomal sorting complex required for transport (ESCRT) complex (Hurley, 2010; Feng et al., 2014; Primadharsini et al., 2020), which is involved in the budding process of enveloped virus.

## Hepatitis E Virus-Open Reading Frame 2 as a Vaccine Candidate

*In vitro* cell culture systems have greatly facilitated the development of both attenuated vaccines and inactive vaccines against a virus. However, available cell culture systems, such as Huh7-S10-3 cells, A549 cells, and HepG2/C3A cells, are not sufficient enough to support large-scale production of HEV viral particles regardless of HEV genotypes (Tanaka et al., 2007; Okamoto, 2011; Berto et al., 2013; Johne et al., 2016; Nan and Zhang, 2016). Thus, HEV vaccine development has to rely on other methods. Fortunately, the capsid protein of HEV shares over 85% identity with all four major HEV genotypes within the species *Orthohepevirus A* infecting humans. Therefore, ORF2 is a good HEV subunit vaccine candidate (Mori and Matsuura, 2011), and has prompted the development of recombinant HEV capsid proteins to be used as subunit vaccines soon after the discovery of HEV (Purdy et al., 1993). Unfortunately, most HEV vaccine programs based on the recombinant HEV capsid were discontinued at the pre-clinical stages due to mixed data or incomplete protection rate, which suggested that the ORF2-based HEV subunit vaccine is more risky than originally speculated (WHO, 2014). A major obstacle that hampered the development of an effective ORF2-based recombinant HEV subunit vaccine is the lack of essential information about proteolytic processing and generation of the HEV capsid protein during the HEV replication cycle.

### Viral Like Particles Vaccine

Earlier report based on the baculovirus expression system suggested that HEV-ORF2 of the HEV-1 Burmese strain can be expressed as the full length 72 kDa form along with a 56.5 kDa form, which is believed to be the proteolyticaly processed shorter product of the full-length protein (McAtee et al., 1996). This observation is further supported by later reports using similar systems which demonstrated that the first 111 and last 52 aa residues were removed from the full ORF2 protein, and the shorter form of ORF2 (aa 112–606) is capable of forming virus-like particles (VLPs) (Li et al., 1997, 2005c; Zhang et al., 1997). The crystal structure obtained from the icosahedral VLPs demonstrates that 60 subunits of truncated ORF2 protein are required for VLPs assembly and truncated ORF2 protein can be further divided into a shell domain (S, aa 129–319), middle domain (M, aa 320–455), and a protruding domain (P, aa 456–606) based on architecture of VLPs (Yamashita et al., 2009). The mutational assay indicated that the P domain is involved in binding to susceptible cells and contains neutralization epitopes (Yamashita et al., 2009). Conversely, another VLP system developed based on HEV-1 (Sar-55 strain) demonstrated confusing results. When expressed in insect cells, translated products of Sar-55 ORF2 demonstrated variable sizes of 72, 63, 56, and 53 kDa (Robinson et al., 1998). Unlike VLPs based on the HEV-1 Burmese strain, only the shortest form of the Sar-55 ORF2 (containing aa 112–578) was able to form a VLP, whereas the 56 kDa Sar-55 ORF2 product (containing aa 112–607 and the P domain) could not (Robinson et al., 1998). The factors involved in the different assemblies of HEV-VLPs remain unclear. Nevertheless, VLPs generated using the 53 kDa fragment from the HEV-1 Sar55 strain could not confer full protective immunity in rhesus macaques after a high-dose challenge, due to the lack of a neutralizing epitope in aa 578–607 (Zhang et al., 2001). Besides VLPs produced using insect cells, a recent study also demonstrated that p495 (aa 112–606 of ORF2) expressed from *E. Coli* can self-assemble into a *T* = 1 VLP (*T*: Triangulation number, a term used for calculating the number of facets per triangular face of an icosahedron virion) (Zheng et al., 2018).

### Subunit Vaccine

In addition to VLPs, recombinantly expressed ORF2 products and truncated ORF2 products were tested for their vaccine potential as well. In China, the first tested recombinant HEV-ORF2 protein was truncated ORF2 (aa 394–607 of ORF2 of a Chinese HEV-1 isolate) expressed in *E. coli*. However, in rhesus macaques, this recombinant vaccine induced protective immunity with three 100-μg doses with Freund’s adjuvant, whereas it was poorly immunogenic in mice if using alum as adjuvant (Li et al., 2005b). However, the immunogenicity of *E. Coli* expressed ORF2 truncation could be improved by further truncating the protein into 239aa (aa368–606 of ORF2) containing the full P domain (Li et al., 2005a, b) and 239aa truncation (designated as HEV239) is claimed to form VLP similar to that of p495 (Wei et al., 2014). Therefore, HEV239 was moved into clinical trials in humans and approved as the only HEV vaccine available all over the world. Meanwhile, there was report employing truncated ORF2 (p179) produced from yeast cells for a potential vaccine candidate as well. This HEV-ORF2 truncation p179 comprising the aa 439–617 based on a HEV-4 ORF2 was tested both in animals experiment and in a phase I human trial (Xu et al., 2016; Cao et al., 2017). HEV-4 p179 acts as monomer, whereas dimerization of p179 is not required for inducing neutralizing antibodies. Moreover, there was also reports suggested that immunization with two doses of the 56 kD Sar-55-ORF2 truncation (which is unable to form a VLP) conferred protection against challenge of either the homologous HEV-1 strain or heterogeneous HEV-2 and HEV-3 strains (Purcell et al., 2003). Furthermore, for a zoonotic HEV-4 with porcine origin, three ORF2-derived peptides (407eptv410, 410vklyts415, and 458psrpf462) were identified as epitopes that partially blocked neutralizing monoclonal antibodies against HEV-4 infection in rabbits, and immunization of tandem fusion peptides containing above epitopes conjugated with keyhole limpet hemocyanin (KLH) in rabbits conferred full protection against this porcine HEV-4 isolate (Chen et al., 2020).

### DNA Vaccine

In addition to recombinant ORF2 subunit vaccines, DNA vaccines based on full or partial HEV-ORF2 sequence were evaluated in cynomolgus macaques but demonstrated mixed results. The first HEV-ORF2 DNA vaccine (designated as pcHEVORF2) contained the full ORF2 sequence of *HEV-1* Burmese strain (Kamili et al., 2002). However, it failed to confer protection in vaccinated animals against challenged of heterologous HEV-2 (Kamili et al., 2002). The second DNA vaccine was liposome-encapsulated DNA containing the ORF2 coding sequence mixed with 20 μg of truncated ORF2 corresponding to a putative neutralizing epitope (aa 458–607) expressed *via E. coli* based on an Indian HEV-1 strain (Arankalle et al., 2009); this demonstrated full protection in macaques against a challenge with 10,000 copies of HEV RNA (Arankalle et al., 2009).

Based on published literature, it appears that the application of HEV-ORF2 as a common vaccine target generates mixed and conflicted outcomes. Although most reports propose that the capsid protein should be the truncated ORF2 protein containing aa 112–606, the assembly of different VLPs by different ORF2 truncations were observed for different HEV genotypes. Also, vaccine antigenicity (defined as induction of neutralizing antibodies) was variable between full-length ORF2 and ORF2 truncations, implying that a deeper investigation of HEV-ORF2 processing and authentic viral particle assembly is required. Nevertheless, the discovery of quasi-enveloped HEV particles also creates new concerns; an earlier study demonstrated that serum HEV virions obtained from HEV patients could infect cells despite the presence of anti-HEV antibodies, since the membrane-wrapped HEV particles are unable to be recognized by anti-ORF2 antibodies (Takahashi et al., 2010). Therefore, more investigation is required to understand HEV-ORF2 processing and the neutralization of quasi-enveloped HEV virions when developing the next generation of HEV vaccines.

## Regulation of Innate Immune Response and Cellular Signaling by Hepatitis E Virus-Open Reading Frame 2

In addition to its role as a structure protein for virion assembly, the HEV-ORF2 protein is involved in the regulation of the host innate immune response. Although less investigated, some reports have suggested that the HEV-ORF2 protein could interfere with the retinoic acid-inducible gene-I (RIG-I) activation, which plays a key role in initiating interferons (IFNs) production and innate immunity as a pattern recognition receptor (Nan et al., 2017a). In an early report, it was demonstrated that the N-terminal arginine-rich motif of ORF2 (of the HEV-3 Kernow-C1 p6 strain) blocked the phosphorylation of interferon regulatory factor 3 (IRF3) *via* interaction with a multiprotein complex consisting of mitochondrial antiviral-signaling protein (MAVS), TANK-binding kinase 1 (TBK1), and IRF3 (Lin et al., 2019). Notably, it appears that the N-terminal segment containing the first 111 aa of ORF2 is responsible for such inhibition (Lin et al., 2019). However, in another report, it demonstrates that full-length ORF2 protein inhibits the response of RIG-I-dependent IFNs induction, whereas the glycosylation and dimerization of the ORF2 protein had no effect on ORF2 mediated antagonism of RIG-I activation (Hingane et al., 2020). Meanwhile, ORF2 protein could antagonize the TLR pathway as well. IFNs activation triggered by adaptor molecules of TLR pathway, such as MAVS, MyD88, and TRIF, could be inhibited by ORF2 except IRF3, implying that ORF2 may act upstream of IRF3 (Hingane et al., 2020). This observation is consistent with a another report suggesting that HEV-ORF2 interacts with the multiprotein complex compromised of MAVS, TBK1, and IRF3 (Lin et al., 2019), but the exact domain or part of ORF2 involved in this inhibition requires further investigation.

NF-κB plays an essential role in host cell survival during infections as well as in the induction of innate immunity besides interferon, such as increasing the production of pro-inflammatory cytokines (Nan et al., 2014). The activation of NF-κB requires the phosphorylation and degradation of IκB, which releases the nuclear localization signal of the NF-κB dimer so that the downstream signal can be activated (Nan et al., 2014). The β-transducin repeat-containing protein (β-TRCP) is a component of the ubiquitination complex which leads to IκBα ubiquitination and promotes NF-κB activation (Liu et al., 2018). It appears that the HEV capsid interacts with β-TRCP to block the ubiquitin–proteasome-mediated degradation of IκB which subsequently inhibits NF-κB activation (Surjit et al., 2012). Moreover, a recent preprint report suggested that the arginine-rich motifs of the N-terminal of ORF2 could serve as a nuclear location signal to direct the nuclear translocation of ORF2 therefore inhibit the downregulation of NF-κB-related signaling as well (Hervouet et al., 2021).

Furthermore, there is a report which demonstrated that the HEV-ORF2 protein could activate the pro-apoptotic gene C/EBP homologous protein (CHOP) as well (John et al., 2011). In Huh7 cells, the HEV-ORF2 protein stimulated the CHOP promoter mainly through AAREs (amino acid response elements) and ERSEs (endoplasmic reticulum stress response elements) (John et al., 2011); it also increased the expression of Hsp72, Hsp70B, and Hsp40. Notably, HEV-ORF2 interacts with Hsp72 and promoted the nuclear accumulation of Hsp72 (John et al., 2011). Taken together, these data suggested that HEV-ORF2 modulates the host innate immune response and cellular signaling to facilitate virus replication.

## Involvement of Hepatitis E Virus-Open Reading Frame 2 Protein in Host Tropism

Since the discovery of interspecies transmission of HEV between swine and humans in the 1990s, zoonotic HEV strains are constantly identified from many mammalian species. Based on the capability to cause zoonotic infection or not, HEV isolates can be classified as human-restrictive HEV isolates (HEV-1 and 2), zoonotic HEV isolates (HEV-3, -4, and potentially -7, and -8), or animal-restrictive HEV isolates (*Orthohepevirus C*). Until now, viral and host factors determining host tropism among different HEV genotypes are still unclear. However, HEV-3- and HEV-4-based reverse genetic systems could be employed to investigate the potential viral determinants for host tropism by swapping genetic segments between infectious clone of different HEV genotypes.

As the viral capsid protein, HEV-ORF2 was initially not considered as a determinant of host tropism due to it being relatively conserved among HEV genotypes infecting humans (Meng, 2016). Since cellular receptors for HEV remain unclear, it is not certain if zoonotic HEV employs the same receptor to infect the host regardless of species. However, an *in vivo* infection experiment based on swapping ORFs using reverse genetic suggested that zoonotic HEV-3- or HEV-4-based chimeric viruses bearing ORF2 originating from HEV-1 are rescuable and viable *in vitro*; however, these chimeric viruses failed to infect or establish a robust infection in swine (Cordoba et al., 2012). Therefore, these data suggested that HEV-ORF2 is involved in HEV-interspecies infection. In a more detailed report, an HEV-3-based chimeric virus bearing partial capsid region spanning aa 456–605 (covered the ORF2-P domain and putative cell receptor-binding region from the corresponding region of HEV-1) failed to enter and infect swine cells (Nguyen et al., 2014), further supporting the conclusion that HEV capsid proteins determine host preference as well. Nevertheless, until cellular receptors for HEV are identified, the link between viral capsid residues and cellular receptor determinants underlying HEV host tropism still requires further investigation. Conversely, an *in vitro* report suggested that HEV-1-based recombinant virus bearing ORF1 from HEV-4 could replicate in porcine kidney cells, whereas the original HEV-1 could not (Chatterjee et al., 2016); this is in agreement with another report indicating that the 5′ NCR and ORF1 are involved in HEV cross-species infection *in vivo* (Feagins et al., 2011). However, a recent report demonstrated that the swapping of ORF1 regions between HEV-1 and HEV-3 generated viable chimeric viruses *in vitro*, but failed to infect piglets *in vivo* (Tian et al., 2020). Therefore, although no direct investigation of ORF3’s role in host tropism was reported, available literature indicated that HEV-ORF2 is a putative determinant of HEV host tropism in addition to ORF1.

## Hepatitis E Virus-Open Reading Frame 2 Protein as a Novel Secretable Factor

### Secreted Forms of Open Reading Frame 2 Dimers

Sequence analysis suggested that full-length HEV-ORF2 contains 660 aa residues and potentially carries N-linked glycans at three potential glycosylation sites (Asn137, Asn310, and Asn562) (Jameel et al., 1996). As glycosylation of the capsid protein in non-enveloped viruses is very rare, it was questionable for a long time whether the HEV-ORF2 protein was truly glycosylated or not, and what biological role would be played by such post-translational modifications in HEV infection and pathogenesis. Although there is evidence supporting the existence of glycosylated ORF2, data from different groups appears inconsistent with each other. On the one hand, identification of a 15 aa ER-directing signal peptide suggested that ORF2 could experience post-translational modifications such as N-linked glycosylation (Jameel et al., 1996; Kurys et al., 2000), where the mutagenesis of the signal peptide in ORF2 changes its subcellular localization (Zafrullah et al., 1999). On the other hand, among all three putative sites (Asn137, Asn310, and Asn562), it appears that Asn-310 could be the major site for glycosylation (Zafrullah et al., 1999); mutations in the first two glycosylation sites prevented virion assembly, whereas mutation of the third site still allowed virion particle formation and RNA encapsulation (Graff et al., 2008). However, these mutagenesis assays still failed to give a clear biological significance of the glycosylation of HEV-ORF2.

In 2018, a new report demonstrated that at least two different forms of HEV-ORF2 protein translation products were detected in HEV-3 Kernow-C1 p6 strain-infected HepG2/C3A cells (Yin et al., 2018). The first HEV-ORF2 was a secreted form of the ORF2 protein (ORF2s) and was translated from the start codon originally thought to initiate translation of the full-length ORF2 (Yin et al., 2018). The ORF2s contained the 15aa signal peptide which directs ORF2s for subsequent glycosylation and the secretion pathway (Yin et al., 2018). The second ORF2 product was a capsid-associated truncated form of ORF2 (ORF2c), which was initiated from an internal AUG start codon (aa16 of ORF2) immediately downstream of signal peptides (Yin et al., 2018). Currently, there is no evidence supports the secretion and glycosylation had occurred in internal initialized ORF2c and a recent study demonstrated that N-glycosylation of ORF2 protein does not play any role in replication and assembly of infectious HEV particles in *HEV-3* KernowC-1 p6 strain (Ankavay et al., 2019). However, these data observed in *HEV-3* appears to be conflict with previous result showed that mutations within any individual glycosylation sites within ORF2 in *HEV-1* Sar55 strain prevented the formation of infectious virus particles without affecting viral RNA replication (Graff et al., 2008). Therefore, glycosylation of capsid-associated ORF2 or such glycosylation is HEV genotype-specific requires further investigation.

The glycosylated ORF2s exists as a dimer which can be secreted into supernatant from HEV-infected cells and is not associated with HEV virions (Yin et al., 2018). However, antigenicity analysis between ORF2s and VLPs using a panel of ORF2-specific monoclonal antibodies (Mabs) suggested that the putative epitopes of HEV capsids predicted to bind with potential cell receptors are lost in dimerized ORF2s, and therefore could not be recognized by corresponding Mabs (Yamashita et al., 2009; Yin et al., 2018). Meanwhile, it is also notable that dimerized ORF2s is unable to bind to susceptible cells therefore does not interfere with HEV virion entry into susceptible cells; however, it does interfere with antibody-mediated viral neutralization of the HEV-3 Kernow-C1 p6 strain in HepG2/C3A cells (Yin et al., 2018). It appears that the dimerized ORF2s does exhibit substantial antigenic overlap with the HEV capsid but could act as a decoy to block the antibody-mediated neutralization of authentic viral particles (Yin et al., 2018). This observation may partially explain why utilizing full-length ORF2 or longer truncations of HEV-ORF2 as vaccine candidates failed to confer effective protection in vaccinated animals against HEV challenge in previous reports, since antigenicity appears to be variable between different HEV-ORF2 forms. Also, since no *in vitro* HEV cell culture system is compatible for all HEV genotypes, whether secretable full-length ORF2 presents in all HEV genotypes or if they are genotype-specific still requires further investigation. Conversely, since dimerization appears to be a universal characteristic for either full length ORF2 or truncated ORF2 such as p239 (Li et al., 2005a; Wei et al., 2018; Yin et al., 2018), it is unclear why truncated ORF2 p239 maintains full antigenicity to evoke a protective humoral response whereas full ORF2s cannot. Explanation for this controversial observation will provide new insight for future HEV vaccine development.

### Potential Modulatory Effects of Secreted Open Reading Frame 2

In addition to interfere with the antibody-mediated viral neutralization by dimerized ORF2s, it would be interesting to elucidate whether dimerized ORF2s could bind with other membrane proteins presented on the cell surface as such scenario might confer ORF2s a cytokine-like function. In *in vitro* experiment, the core protein of the Hepatitis C virus (HCV), the major component of HCV nucleocapsids, could be secreted from either HCV-infected cells or hepatoma cells transfected with HCV-core encoding plasmid (Maillard et al., 2001; Sansonno et al., 2003). This observation is consisted with the fact that HCV-core protein could be detected in large amounts in HCV patients’ serum (Maillard et al., 2001; Sansonno et al., 2003). Meanwhile, truncation analysis suggests that that the C-terminal domain of the HCV-core protein is essential for extracellular secretion (Choi et al., 2005). Notably, extracellular HCV-core protein binds with cellular receptors such as complement receptor gC1qR to inhibit T-lymphocyte proliferation (Sabile et al., 1999; Kittlesen et al., 2000), which demonstrates immune-modulator function. Moreover, extracellular HCV-core protein activates STAT3 in human monocytes/macrophages/dendritic cells *via* an IL-6 autocrine pathway (Tacke et al., 2011). Activation of IL-6 autocrine pathway plays a critical role in the modulation of inflammatory responses by APCs and potentially impairs T cell response during HCV infection (Tacke et al., 2011). Besides the HCV-core protein, the similar scenario is observed in hepatitis B virus (HBV) as well. The naked capsids of HBV, which is composed by 180 or 240 HBV core proteins, are released from HBV-infected cells as well and altered host immune response (Lazdina et al., 2001; Chou et al., 2015). Therefore, since the presence of large amounts of non-virion-associated ORF2 protein in HEV-infected patient serum is frequently reported (Yin et al., 2018), it is possible that secretable HEV-ORF2 proteins may play a role in modulating anti-HEV immunity similar to that in HCV or HBV.

Meanwhile, another report using the same HEV strain (Kernow-C1 p6 strain), but in a subclone of the PLC/PRF/5 cell line, demonstrated that there are three forms of ORF2 generated during the HEV replication cycle, with two glycosylated forms of ORF2 associated with the secretable pathway and a non-glycosylated form associated with the infectious virion (Montpellier et al., 2018). Regarding the two glycosylated forms of ORF2, one is the full-length ORF2 whereas the other one appears to be cleaved by an unknown protease but is still secretable as the full-length ORF2 protein (Montpellier et al., 2018). However, the exact cleavage sites, characteristics, and biological function of the shorter secretable ORF2 protein remains unclear. It is also unknown if dimerization could occur in the shorter secretable ORF2 protein as well. Taken together, available literature indicates that HEV-ORF2 undergoes extensive post-translational modification and protease processing, which generates different forms of ORF2 protein involved in unique steps of viral replication and assembly, as well as other biological functions beyond these.

## Conclusion and Future Perspectives

More than two decades have passed since the discovery of HEV; geographical distribution of HEV is expanding, therefore HEV is no longer restricted to developing countries. Although tremendous efforts have been dedicated to understanding this virus, only one subunit vaccine based on its capsid protein is available in China and our understanding toward function of HEV proteins remains limited. It appears that more unexpected functions of HEV-ORF2 are revealed from ongoing research, and HEV is much more complicated than scientists originally expected.

HEV-ORF2 was previously thought to only participate in the assembly of the mature virion and HEV was originally classified as a non-enveloped virus. Nevertheless, on the one hand, the discovery of the quasi-enveloped HEV virion challenged the basic dogma of the virus classification system based on the presence or absence of an envelope in virion structure. On the other hand, secretory form of ORF2 also prompted researchers to investigate more functions of the HEV capsid protein beyond being a viral antigen for a vaccine development. Currently, available literature demonstrates that HEV-ORF2 can be translated into different forms that undergo various post-translational processing and perform different functions, such as host innate immune response and cell signaling regulation, determination of host tropism, and participation in HEV pathogenesis. These advances can guide further studies to reveal more functions of the HEV-ORF2 proteins and HEV pathogenic factors which can promote the development of effective therapeutics and provide new research directions for the hepatitis E virus.

## Author Contributions

All authors listed have made a substantial, direct and intellectual contribution to the work, and approved it for publication.

## Conflict of Interest

The authors declare that the research was conducted in the absence of any commercial or financial relationships that could be construed as a potential conflict of interest.

## Publisher’s Note

All claims expressed in this article are solely those of the authors and do not necessarily represent those of their affiliated organizations, or those of the publisher, the editors and the reviewers. Any product that may be evaluated in this article, or claim that may be made by its manufacturer, is not guaranteed or endorsed by the publisher.
